# Neurobehavioral Association Between Macroscopic Network Decoupling and Reduced Eating Impulsivity: A Longitudinal fMRI Study in Bulimia Nervosa

**DOI:** 10.1002/cns.71129

**Published:** 2026-09-01

**Authors:** Yulong Jia, Weihua Li, Demin Kong, Fengxia Yu, Guowei Wu, Yiling Wang, Jiani Wang, Zhenchang Wang, Lirong Tang, Peng Zhang

**Affiliations:** ^1^ Department of Radiology, Beijing Friendship Hospital Capital Medical University Beijing China; ^2^ Department of Radiology Zibo Central Hospital Zibo Shandong China; ^3^ CAS Key Laboratory of Behavioral Science, Institute of Psychology Chinese Academy of Sciences Beijing China; ^4^ Department of Radiology The Affiliated Hospital of Qingdao University Qingdao China; ^5^ Precision and Intelligence Medical Imaging Lab, Beijing Friendship Hospital Capital Medical University Beijing China; ^6^ The National Clinical Research Center for Mental Disorders & Beijing Key Laboratory of Mental Disorders, Beijing Anding Hospital Capital Medical University Beijing China

**Keywords:** bulimia nervosa, eating impulsivity, fluoxetine, functional connectivity, salience network

## Abstract

**Background:**

Fluoxetine is an established pharmacological intervention for bulimia nervosa (BN); however, the macroscopic functional network remodeling underlying its efficacy in suppressing eating impulsivity remains poorly understood.

**Methods:**

Fifty‐eight patients with BN and 58 healthy controls assessed at both baseline and follow‐up underwent resting‐state functional magnetic resonance imaging (rs‐fMRI). Network‐Based Statistic (NBS) analysis with edgewise paired comparisons was used to identify whole‐brain connectivity changes in patients with BN. The resulting BN‐derived component was subsequently examined in both groups using a Group × Time analysis. Exploratory brain‐behavior associations within BN were examined using Spearman and partial Spearman correlations.

**Results:**

During follow‐up, patients with BN showed significant improvements in depressive symptoms and externally driven eating behavior. Within‐group NBS analysis identified a 23‐edge component showing reduced functional connectivity in BN at follow‐up, with network‐level family‐wise error correction (*p* < 0.05). This component primarily involved dorsal attention, ventral attention/salience, and somatomotor networks. Whole‐brain NBS analysis in healthy controls did not identify a significant longitudinal component. When mean connectivity of the BN‐derived component was extracted in both groups, connectivity decreased more strongly in BN than in healthy controls (Group × Time *β* = −0.053, *p* = 0.027).

**Conclusions:**

The follow‐up period in patients with BN was accompanied by clinical improvement and reduced functional connectivity among attention‐, salience‐, and sensorimotor‐related systems. These findings provide preliminary evidence of treatment‐period‐associated functional network reconfiguration in BN.

**Trial Registration:**

ChiCTR2200066885

## Graphical abstract

Fifty‐eight patients with bulimia nervosa underwent longitudinal resting‐state fMRI at baseline and follow‐up during fluoxetine treatment, with 58 healthy controls serving as a longitudinal reference cohort. Network‐Based Statistic analysis identified reduced connectivity within an attention–salience–sensorimotor component, with follow‐up connectivity in patients shifting toward the healthy‐control reference range. Exploratory analyses suggested that greater network change was associated with reduced externally driven eating behavior.

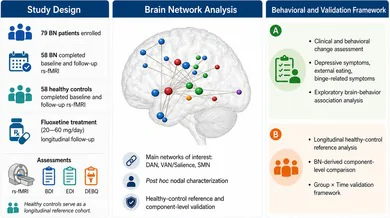

## 1 Introduction

BN is a severe eating disorder characterized by recurrent episodes of binge eating accompanied by inappropriate compensatory behaviors, such as self‐induced vomiting or excessive exercise [1]. In addition to substantial metabolic disturbances and high rates of psychiatric comorbidity, BN is marked by heightened sensitivity to external food‐related cues, often resulting in pronounced cue‐induced eating impulsivity [2, 3, 4]. This externally driven impulsivity is highly recognized as a prominent trigger for binge‐eating episodes and serves as a significant barrier to sustained recovery, frequently contributing to the high relapse rates observed following conventional psychological interventions [5]. Accordingly, elucidating the neural mechanisms underlying this maladaptive responsiveness to environmental cues remains a central challenge in both clinical management and neuroscientific research on BN.

SSRIs, particularly fluoxetine, represent the only pharmacological treatment approved by the U.S. Food and Drug Administration (FDA) for BN [6]. Randomized controlled trials have demonstrated that fluoxetine, especially at higher doses (e.g., 60 mg/day), reduces binge‐eating frequency and compensatory behaviors, while also improving comorbid affective symptoms [7, 8]. Despite its established clinical efficacy, the neural mechanisms through which fluoxetine exerts its therapeutic effects remain incompletely understood [9]. While its primary molecular action involves inhibition of the serotonin transporter and subsequent increases in synaptic serotonin levels, how these changes translate into large‐scale functional brain network reorganization is not well characterized [10, 11]. Bridging this gap is essential for a more comprehensive understanding of SSRI effects in eating disorders.

Recent advances in rs‐fMRI have highlighted the role of large‐scale brain networks in the pathophysiology of BN. Emerging models suggest that abnormal eating behavior may be associated with altered interactions among attention, salience, and sensorimotor systems [12, 13]. In particular, the Ventral Attention Network (VAN), implicated in the detection of behaviorally relevant stimuli, and the Dorsal Attention Network (DAN), involved in goal‐directed attentional control, are thought to play complementary roles in regulating responses to external cues [14, 15]. Dysregulated interactions between these systems have been proposed to contribute to attentional biases toward salient stimuli, including food‐related cues in individuals with BN [16]. In addition, the Somatomotor Network (SMN) has been suggested to contribute to the translation of cognitive‐affective states into behavioral responses, potentially linking craving‐related processes with the execution of eating behavior [17]. Together, these observations point to the possibility that altered coordination among attention, salience, and sensorimotor networks may underlie aspects of cue‐induced eating impulsivity in BN.

Although cross‐sectional studies have identified functional alterations within these networks, longitudinal investigations examining how pharmacological interventions influence large‐scale network organization in BN remain limited [18]. To address this gap, the present study employed a longitudinal rs‐fMRI design combined with NBS analysis to examine longitudinal changes in whole‐brain functional connectivity in patients with BN during fluoxetine treatment. In addition, a longitudinal healthy‐control reference cohort was included to evaluate whether the observed connectivity changes could be attributed to non‐specific temporal drift or repeated‐scanning effects. Based on prior theoretical and empirical work, we first examined whether the treatment period in BN was accompanied by altered functional connectivity within networks implicated in attention, salience processing, and sensorimotor control. Second, we tested whether the BN‐derived network changes were more pronounced than longitudinal changes observed in healthy controls. Finally, we explored whether network‐level changes within BN were associated with improvements in eating‐related behavioral measures, while accounting for potential physiological and temporal confounds. These analyses were intended to provide preliminary evidence regarding the relationship between large‐scale network dynamics and clinical improvement during fluoxetine treatment in BN.

## 2 Materials and Methods

### 2.1 Participants

The Institutional Review Board of Beijing Friendship Hospital, Capital Medical University approved this single‐center prospective cohort study (Approval Number: 2021‐P2‐052‐01). The study protocol was aligned with the Declaration of Helsinki guidelines. All patients signed written informed consent forms after being fully briefed on the nature, processes, and risks of the research. Patients with BN were recruited from the outpatient clinical services of Beijing Anding Hospital, Capital Medical University. Patients with BN were independently recruited by two psychiatrists specializing in eating disorders. Diagnoses were established using the Mini‐International Neuropsychiatric Interview (MINI) version 7.0.2 [19], a brief, structured diagnostic tool based on the criteria outlined in the Diagnostic and Statistical Manual of Mental Disorders, Fifth Edition (DSM‐5) [20]. Exclusion criteria for the BN cohort included: (1) current major psychiatric comorbidities (e.g., schizophrenia, bipolar, dissociative, or substance use disorders), excluding depression or anxiety; (2) complex polypharmacy or clinically relevant deviations from the standardized fluoxetine monotherapy protocol during follow‐up, including discontinuation of fluoxetine, switching to or adding another psychotropic medication, or prolonged dose reduction because of intolerance or adverse effects; (3) poor treatment adherence (< 80%). All patients underwent resting‐state functional magnetic resonance imaging (rs‐fMRI) and comprehensive clinical and behavioral assessments at baseline and follow‐up during the standardized fluoxetine treatment period. Clinical evaluations included the Beck Depression Inventory (BDI), Eating Disorder Inventory (EDI), and the Dutch Eating Behavior Questionnaire (DEBQ).

A longitudinal healthy‐control reference cohort was also included to evaluate non‐specific longitudinal or repeated‐scanning effects. Healthy controls were recruited from hospital staff and community volunteers and were frequency‐matched to the BN group for age, sex, and educational background. Healthy controls had no history of eating disorders, major psychiatric disorders, neurological disorders, or psychoactive medication use. They underwent the same rs‐fMRI protocol at baseline and follow‐up but did not receive fluoxetine or other study‐related treatment. Only participants with usable imaging data at both time points and acceptable head‐motion quality control were included in the final longitudinal analyses.

Initially, 79 patients with BN and 60 healthy controls were included in the longitudinal neuroimaging analyses.

### 2.2 Standardized Pharmacological Intervention

The pharmacological protocol was strictly implemented in accordance with the American Psychiatric Association (APA) practice guidelines for eating disorders and the U.S. FDA recommendations for BN [21, 22]. All enrolled patients received a standardized monotherapy of fluoxetine, managed by board‐certified psychiatrists who were blinded to the neuroimaging data.

Fluoxetine was administered orally once daily in the morning. To minimize early adverse effects (e.g., gastrointestinal distress, agitation), treatment was initiated at a dose of 20 mg/day. Based on individual tolerance, the dosage was systematically titrated over a 2–4‐week period to the target therapeutic dose of 60 mg/day, which is the established optimal dosage for effectively reducing binge‐eating and compensatory behaviors in BN [23].

Clinical monitoring and medication adherence evaluations (via pill counts and self‐reports) were conducted monthly. The pharmacological intervention would be immediately terminated, and the patient withdrawn from the study, under any of the following conditions: (1) occurrence of intolerable adverse effects (e.g., severe allergic reactions, suspected serotonin syndrome); (2) emergence of severe psychiatric comorbidities, such as active suicidal ideation or manic episodes; (3) positive pregnancy test; or (4) voluntary withdrawal of consent.

### 2.3 MRI Data Acquisition

To ensure data reliability, participants adhered to standardized pre‐scan conditions. Caffeine and alcohol were restricted for 72 h prior to imaging to prevent metabolic and hemodynamic confounds [24, 25]. After a minimum 4‐h fast, subjective hunger was measured using a 10‐point Visual Analog Scale (VAS; 0 = not hungry, 10 = extremely hungry). To avoid brain activity alterations driven by extreme hunger or fullness [26], only subjects scoring between 3 and 7 were scanned as planned; those outside this window were rescheduled.

Imaging data were obtained on a 3.0‐T MRI system (Prisma, Siemens, Erlangen, Germany) equipped with a 64‐channel head coil. Initially, standard axial T2 sequences were employed to rule out obvious brain abnormalities. Subsequently, an echo‐planar imaging (EPI) sequence was applied to capture functional data (TR = 2000 ms, TE = 30 ms, flip angle = 90°, matrix = 64 × 64, FOV = 224 × 224 mm [2], slice thickness/gap = 3.5/1 mm, 33 slices, 240 time points). All subjects confirmed they maintained wakefulness during the entire experiment. Prior to MRI scanning, demographic data were collected, and all participants completed a minimum of 4 h fast.

### 2.4 fMRI Preprocessing and Brain Network Extraction

To ensure rigorous data quality and reproducible denoising, the preprocessing and post‐processing of the functional MRI data were executed within a Windows Subsystem for Linux (WSL) environment [WSL 2, Version 2.6.3.0]. The pipeline combined fMRIPrep [Version 25.2.5] for minimal preprocessing and XCP‐D [Version 26.0.3] (eXactly Computable Project—Denoising) for robust confound regression and network extraction. The meticulous steps are detailed as follows:

(1) Volumetric Preprocessing and Coregistration: Initial preprocessing was performed via fMRIPrep. This included brain extraction, spatial normalization of anatomical images to the MNI152NLin6Asym standard space utilizing Advanced Normalization Tools (ANTs), and tissue segmentation. Functional data were subjected to motion correction (FSL), slice‐timing correction, and co‐registered to the corresponding structural images using FreeSurfer's Boundary‐Based Registration (BBR) algorithm. (2) Framewise Displacement and Scrubbing: To mitigate the artifactual effects of subject head motion, framewise displacement (FD) was computed for each volume. Volumes exceeding a strict threshold (FD > 0.2 mm) were flagged as motion outliers prior to denoising to prevent motion‐related spikes from skewing the regression model. (3) Despiking: Prior to confound regression, artifactual spikes and extreme values within the BOLD timeseries were truncated and mitigated using a time‐series interpolation method (despiking), enhancing the temporal stability of the signal. (4) Nuisance Regression: A comprehensive 36‐parameter confound regression strategy (36P) was applied to the data via XCP‐D to regress out physiological and motion‐related noise. The confound regressors encompassed 6 head motion parameters, the global signal (GSR), the mean white matter (WM) signal, and the mean cerebrospinal fluid (CSF) signal, alongside their 9 temporal derivatives and 18 quadratic expansions. (5) Band‐pass Filtering: Following regression, the residual BOLD timeseries underwent temporal band‐pass filtering (0.01–0.08 Hz). This step effectively isolated the low‐frequency spontaneous BOLD fluctuations of interest while filtering out high‐frequency physiological noise (e.g., cardiac and respiratory cycles) and low‐frequency scanner drift. (6) Spatial Smoothing: The denoised functional images were spatially smoothed using a 6.0 mm full‐width at half‐maximum (FWHM) Gaussian kernel to improve the signal‐to‐noise ratio and account for residual inter‐subject anatomical variability. (7) Parcellation and Network Connectivity: Following rigorous denoising, Regional time series were extracted using the 4S256 atlas (Schaefer Supplemented with Subcortical Structures) from the PennLINC AtlasPack, with the cortical parcellation based on Schaefer2018v0143 and assigned to the Yeo 17‐network system. A comprehensive whole‐brain functional connectivity (FC) matrix (256 × 256) was constructed for each participant by calculating Pearson correlation coefficients between the averaged timeseries of all parcel pairs. (8) Quality Control: Given the susceptibility of resting‐state fMRI to head‐motion confounds, head motion was quantified using FD. Volumes with FD > 0.2 mm were flagged as high‐motion volumes for censoring in XCP‐D. In addition, participants with excessive head motion, defined as mean FD > 0.2 mm across the scan, were excluded from the final statistical analyses. The number of censored volumes and mean FD were summarized for each group and time point, and longitudinal differences in motion were evaluated using Wilcoxon signed‐rank tests. Thus, a total of 58 patients with BN were included in the subsequent analyses.

### 2.5 Network‐Based Statistic and Post Hoc Nodal Characterization

Whole‐brain longitudinal changes in functional connectivity were assessed using the NBS [NBS toolbox version 1.2] in MATLAB R2021b. For each edge, a paired‐samples *t*‐test was performed to compare baseline and follow‐up connectivity values within the same participants. Connectivity increases and decreases were tested separately. Connected components were defined using a primary threshold of uncorrected *p* < 0.001, and component‐level significance was assessed using 10,000 permutations. Statistical significance was defined as family‐wise error‐corrected *p* < 0.05.

After identifying the significant NBS component, post hoc nodal characterization was performed to describe the distribution of the component. Nodal strength was summarized for the right temporo‐occipito‐parietal parcel (Node 148) and the right somatomotor parcel (Node 118), which showed prominent involvement within the BN‐derived NBS component. Because these post hoc nodal comparisons were restricted to two parcels, Bonferroni correction was applied, with significance defined as *p* < 0.025.

### 2.6 Statistical Analysis

To evaluate potential selective follow‐up bias, baseline demographic and clinical characteristics were compared between participants who completed the follow‐up assessment and those who dropped out. Independent two‐sample *t*‐tests were used for continuous variables, and chi‐square or Fisher's exact tests were used for categorical variables as appropriate.

All primary analyses were conducted using available paired observations for each outcome. For each longitudinal variable, change scores were calculated as follows: follow‐up minus baseline values. Changes in clinical symptom severity following treatment, as well as baseline‐to‐follow‐up changes in nodal strength of the post hoc nodal parcels identified from the NBS component, were evaluated using paired‐samples *t*‐tests.

Brain‐behavior associations were examined using non‐parametric Spearman's rank correlations between longitudinal changes in network‐level functional connectivity and changes in eating‐related behavioral measures, including ΔDEBQ‐External and ΔEDI‐BN. Partial Spearman correlations were further performed as sensitivity analyses to assess whether these associations were influenced by potential covariates, including body weight change, variation in follow‐up interval, and depressive symptom change. These covariates were examined separately and, where appropriate, jointly. Because these brain‐behavior analyses were exploratory, nominal *p* values are reported, and the findings were interpreted cautiously.

For multiple‐comparison control, connectome‐wide functional connectivity analyses were corrected using NBS, which provides permutation‐based network‐level family‐wise error correction for connected components. For the post hoc nodal‐strength analysis restricted to two nodal parcels, Bonferroni correction was applied using a corrected significance threshold of *p* < 0.025. Statistical significance for confirmatory analyses was defined as two‐tailed *p* < 0.05 after the correction procedure specified for each analysis.

## 3 Results

### 3.1 Demographic and Clinical Characteristics

Initially, 79 patients with BN were enrolled and completed the baseline clinical and neuroimaging assessments. During the longitudinal follow‐up period, 21 patients were excluded from the final analysis for the following reasons: loss to follow‐up or voluntary withdrawal of consent (*n* = 12), discontinuation of medication due to intolerable adverse effects (*n* = 4), and excessive head motion artifacts during the follow‐up MRI scan (*n* = 5). Consequently, a total of 58 patients with BN (55 females and 3 males; mean age: 25.29 ± 5.73 years) were included in the final longitudinal analysis. The planned course of fluoxetine treatment was 6 months; however, the follow‐up clinical and MRI assessments included in the present analyses were conducted 122–140 days after baseline. Notably, the markedly skewed female‐to‐male ratio in our final sample closely mirrors the established epidemiological prevalence and demographic distribution of bulimia nervosa [27]. Among the 60 healthy controls initially included, two were excluded because of excessive head motion during the follow‐up MRI scan. Therefore, the final healthy‐control cohort included 58 participants. The healthy controls were all female and had a mean age of 24.55 ± 2.15 years. The BN and healthy‐control groups did not differ significantly in age, sex distribution, height, weight, body mass index, or years of education (all *p* > 0.05; Table 1). Fasting duration was slightly longer in the BN group than in healthy controls (8.84 ± 4.34 vs. 7.13 ± 3.41 h, *p* = 0.020). No participants in either group reported alcohol use or smoking. At baseline, the median duration of illness in the BN group was 33.0 months, with an interquartile range of 14.0–96.0 months (Table 1).

**TABLE 1:** Demographic characteristics of patients with bulimia nervosa and healthy controls.

Variable	BN baseline (*n* = 58)	BN follow‐up	HC (*n* = 58)	*T* value	*p*
Female/male, *n*	55/3	—	58/0		0.243
Age, years	25.29 ± 5.73	—	24.55 ± 2.15	0.92	0.359
Height, cm	164.14 ± 6.92	—	162.64 ± 4.73	1.36	0.176
Weight, kg	56.37 ± 10.23	—	53.93 ± 4.98	1.63	0.107
BMI, kg/m^2^	20.84 ± 2.98	—	20.40 ± 1.83	0.94	0.347
Education, years	16.53 ± 1.95	—	16.97 ± 2.35	−1.07	0.285
Fasting duration, h	8.84 ± 4.34	—	7.13 ± 3.41	2.36	0.020
Duration of illness, m	33.0 (14.0–96.0)	—	NA	—	—
Binge‐eating episodes/week	6.33 ± 5.38	2.62 ± 3.68	—	−5.07	**< 0.001**
Compensatory behaviors/week	1.27 ± 1.93	0.52 ± 1.42	—	−3.04	**0.004**

*Note:* Continuous variables are presented as mean ± SD except duration of illness, which is presented as median (interquartile range). Group comparisons for continuous variables used Welch independent‐samples *t*‐tests, and sex distribution was compared using Fisher's exact test. Longitudinal eating‐symptom changes in the BN group were evaluated using paired‐samples *t*‐tests based on available paired observations; frequency values containing ranges were converted to midpoint values before analysis. Bold values indicate statistical significance at *p* < 0.05.

Abbreviations: BMI, body mass index; BN, bulimia nervosa; HC, healthy controls; NA, not applicable.

Between baseline and follow‐up, patients with BN showed reductions in eating‐related symptom frequency. Binge‐eating episodes decreased from 6.33 ± 5.38 to 2.62 ± 3.68 episodes per week (*p* < 0.001), and compensatory behaviors decreased from 1.27 ± 1.93 to 0.52 ± 1.42 episodes per week (*p* = 0.004; Table 1).

### 3.2 Longitudinal Clinical and Behavioral Changes During Treatment

At the follow‐up assessment, patients with BN exhibited significant improvements across multiple clinical domains. Depressive symptoms were markedly reduced, as indicated by a decrease in BDI scores (Mean Δ = −7.37, *t* = −6.25, *p* < 0.001). Eating‐related behavioral measures also showed significant improvements. External eating tendencies decreased significantly (DEBQ‐External: Mean Δ = −3.39, *t* = −3.85, *p* < 0.001). Similarly, emotional eating and bulimic symptom severity (EDI‐BN) were significantly reduced (both *p* < 0.001; Table 2).

**TABLE 2:** Longitudinal changes in clinical and behavioral measures following fluoxetine intervention.

Clinical variable	Mean Δ	SD	*t*‐Statistic	*p*
DEBQ‐restraint	−5.76	6.17	−7.225	**< 0.001**
DEBQ‐emotional	−9.86	11.81	−6.471	**< 0.001**
DEBQ‐external	−3.39	6.81	−3.853	**0.0003**
DEBQ‐total	−19.24	18.76	−7.945	**< 0.001**
BDI‐21	−7.37	9.13	−6.251	**< 0.001**
EDI‐BN	−10.33	7.87	−10.169	**< 0.001**
EAT‐26	−16.6	12.95	−9.928	**< 0.001**
SAS‐20	−5.83	14.83	−3.044	**0.0035**
EDI‐DT	−7.06	6.06	−9.03	**< 0.001**
EDI‐BD	−3.02	7.36	−3.181	**0.0023**
EDI‐total	−20.08	15.65	−9.94	**< 0.001**
Weight (kg)	1.18	4.27	2.141	**0.0364**
Fasting, h	−1.28	5.69	−1.746	0.086
VAS	0.36	3.64	0.77	0.4443

*Note:* Data represent the mean longitudinal change (Mean Δ, follow‐up minus baseline) and standard deviation (SD) for clinical, behavioral, and physiological variables measured over the follow‐up period in patients with bulimia nervosa (*n* = 58). Statistical significance was assessed using paired‐samples *t*‐tests. Bold values indicate statistical significance at *p* < 0.05.

Abbreviations: BDI‐21, beck depression inventory; DEBQ, Dutch Eating Behavior Questionnaire; EAT‐26, eating attitudes test; EDI‐BD, eating disorder inventory‐body dissatisfaction; EDI‐BN, eating disorder inventory‐bulimia nervosa; EDI‐DT, eating disorder inventory‐drive for thinness; SAS‐20, self‐rating anxiety scale; VAS, visual analogue scale (subjective hunger).

### 3.3 BN‐Derived Functional Connectivity Component Identified by NBS

**FIGURE 1:**
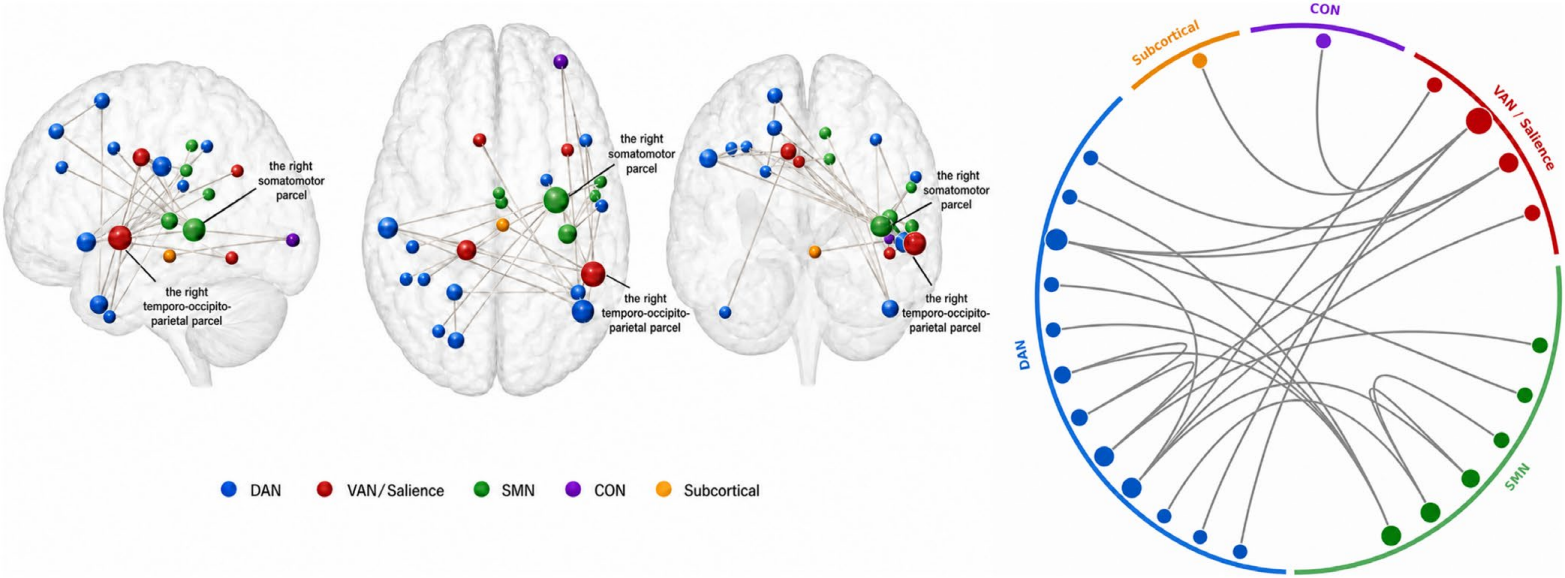
Spatial and circular representation of the BN‐derived NBS component. Surface renderings and the circular connectogram illustrate the 23‐edge component identified by Network‐Based Statistic analysis of paired baseline and follow‐up functional connectivity data within the BN group. For each edge, a paired‐samples *t*‐test was performed between the two time points, and connectivity increases and decreases were tested separately. The displayed component represents functional connectivity decreases from baseline to follow‐up (primary threshold: Uncorrected *p* < 0.001; network‐level FWE‐corrected *p* < 0.05; 10,000 permutations). No significant component was identified for connectivity increases. Nodes are colored according to network affiliation: Dorsal attention network (DAN, blue), ventral attention/salience network (VAN/salience, red), somatomotor network (SMN, green), cingulo‐opercular/control network (CON, purple), and subcortical system (orange). Node size reflects within‐component degree. Right temporo‐occipito‐parietal parcel within the salience/ventral attention network (Node 148) and right somatomotor parcel (Node 118), which showed prominent involvement in the post hoc nodal characterization, are labeled in the surface renderings. The circular connectogram provides a network‐level summary of the same NBS component, showing that the suprathreshold component edges were mainly distributed across DAN, VAN/salience, and SMN, with limited involvement of CON and subcortical regions.

NBS analysis identified a connected sub‐network comprising 23 edges that exhibited significant reductions in functional connectivity following treatment (FWE‐corrected *p* < 0.05; Table 3; Figure 1).

**TABLE 3:** Macroscopic statistical summary of the significantly decoupled sub‐network following fluoxetine intervention.

Interacting network pair	No. of significant edges	*t*‐value	Key anatomical regions involved
DAN ↔ VAN	8	8.37	PPC, mPFC, TOPJ
DAN ↔ SMN	7	8.11	PPC, somatosensory/motor cortex
VAN ↔ SMN	3	8.1	mPFC, somatosensory/motor cortex
DAN ↔ CON/subcortical	5	7.41	PPC, precentral ventral cortex, thalamus

*Note:* This table aggregates the 23 edges exhibiting significant connectivity reductions from baseline to follow‐up (NBS, FWE‐corrected *p* < 0.05). Edges are grouped by network pairs, with the *t*‐value representing the peak statistical strength within each pair.

Abbreviations: CON, cingulo‐opercular network; DAN, dorsal attention network; mPFC, medial prefrontal cortex; PPC, posterior parietal cortex; SMN, somatomotor network; TOPJ, Temporo‐Occipito‐Parietal Junction; VAN, ventral attention/salience network.

**FIGURE 2:**
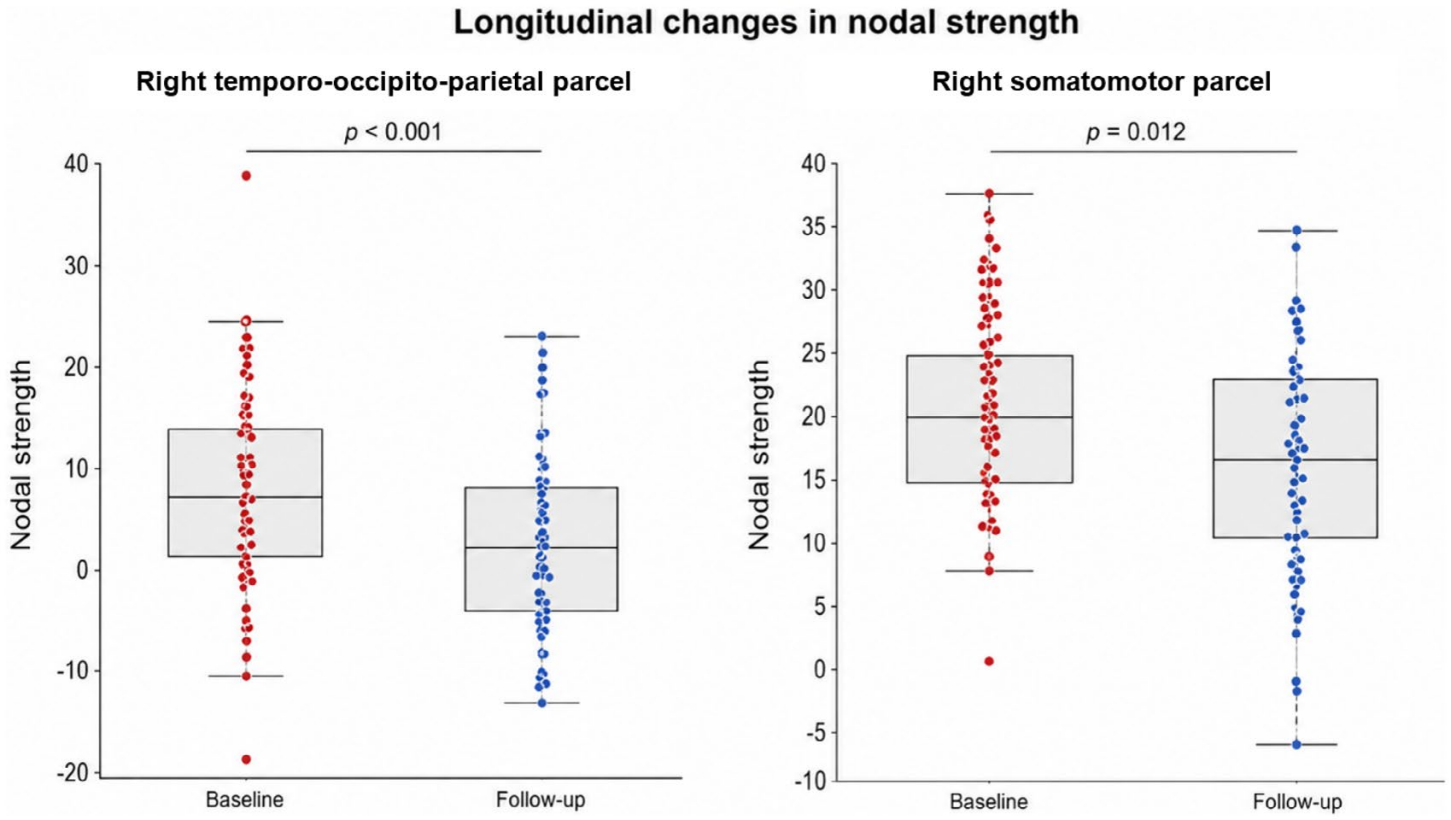
Longitudinal changes in nodal strength of the two post hoc nodal parcels within the BN‐derived NBS component. Boxplots with overlaid individual data points show nodal strength at baseline and follow‐up in the BN group for the right temporo‐occipito‐parietal parcel (Node 148) and the right somatomotor parcel (Node 118). Both parcels showed reduced nodal strength at follow‐up relative to baseline. *p* values were obtained using paired‐samples *t*‐tests. The two post hoc nodal comparisons were interpreted using a Bonferroni‐corrected significance threshold of *p* < 0.025. BN, bulimia nervosa; NBS, network‐based statistic; SMN, somatomotor network; VAN/salience, ventral attention/salience network.

This sub‐network primarily involved connections between the DAN, VAN, and SMN, indicating a pattern of decreased connectivity across attention‐ and sensorimotor‐related systems. In post hoc nodal characterization, reductions in nodal strength were observed in the right temporo‐occipito‐parietal parcel (Node 148, *p* = 0.0002) and the right somatomotor parcel (Node 118, *p* = 0.012; Table 4; Figure 2).

**TABLE 4:** Location of the two post hoc nodal parcels within the BN‐derived NBS component.

Node ID	Network	Anatomical description	MNI center‐of‐mass coordinates, *x*/*y*/*z*	Degree in NBS component
148	VAN/salience	Right temporo‐occipito‐parietal parcel	(56.9, −45.3, 9.5)	5
118	SMN	Right somatomotor parcel	(38.3, −13.4, 14.5)	4

*Note:* Coordinates represent parcel center‐of‐mass locations in MNI152NLin6Asym space based on the Schaefer 4S256 parcellation.

Abbreviations: NBS, network‐based statistic; SMN, somatomotor network; VAN/salience, ventral attention/salience network.

### 3.4 Healthy‐Control Reference Analysis of the BN‐Derived NBS Component

To contextualize the BN‐derived NBS component relative to a healthy longitudinal reference, we extracted the mean FC of the 23‐edge component in both patients with BN and healthy controls at baseline and follow‐up. The BN group showed a marked decrease in component‐level mean FC over time (BN ΔFC = −0.140 ± 0.112, *p* < 0.001). The healthy‐control group also showed a smaller reduction within the same component (HC ΔFC = −0.087 ± 0.145, *p* < 0.001).

**FIGURE 3:**
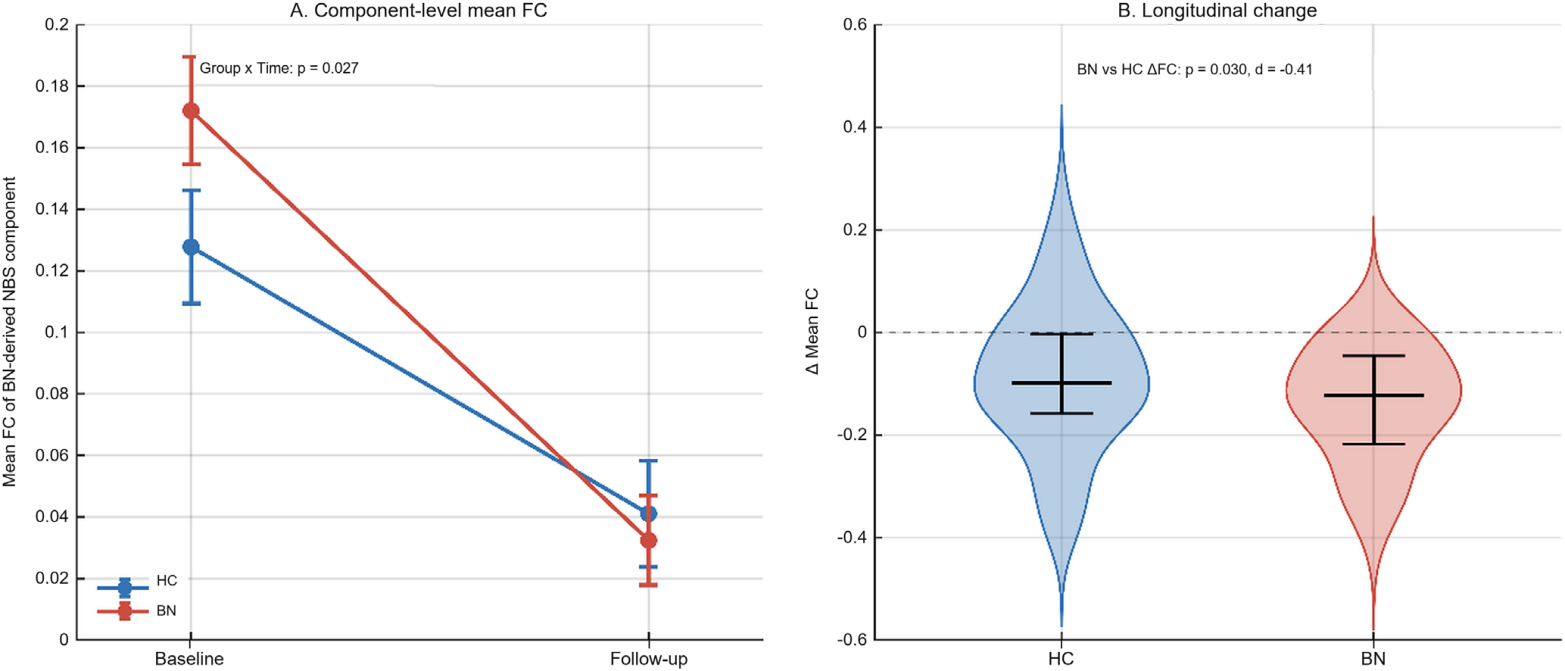
Mean functional connectivity of the BN‐derived NBS component relative to the healthy‐control reference level. (A) Mean functional connectivity across the 23 BN‐derived NBS edges at baseline and follow‐up in patients with BN and healthy controls. At baseline, patients with BN showed elevated component‐level connectivity relative to healthy controls. At follow‐up, connectivity within this component decreased in BN and shifted toward the healthy‐control reference level. (B) Distribution of longitudinal changes in mean FC, defined as follow‐up minus baseline. The Group × Time result is shown as a secondary longitudinal reference analysis. Error bars indicate SEM.

At the whole‐brain level, however, paired NBS analysis in the 58 healthy controls did not identify any significant connected component surviving network‐level FWE correction in either direction of longitudinal change. A secondary component‐level longitudinal comparison showed a significant Group × Time interaction (*β* = −0.053, *p* = 0.027; Table 5; Figure 3).

**TABLE 5:** Healthy‐control reference analysis of mean FC within the BN‐derived 23‐edge NBS component.

Analysis	Contrast	Estimate	*p*
Paired longitudinal change	BN	Δ FC = −0.140 ± 0.112	**< 0.001**
Paired longitudinal change	HC	Δ FC = −0.087 ± 0.145	**< 0.001**
Between‐group comparison	BN Δ FC vs. HC Δ FC	Δ FC = −0.053	**0.030**
Mixed‐effects model	Group × Time	*β* = −0.053	**0.027**

*Note:* ΔFC represents follow‐up minus baseline mean functional connectivity within the BN‐derived 23‐edge NBS component; negative values indicate decreased connectivity over time. Paired changes are shown as mean ± SD. The between‐group comparison and mixed‐effects Group × Time term are presented as secondary component‐level reference analyses to contextualize the BN‐derived longitudinal change relative to healthy controls. Bold values indicate statistical significance at *p* < 0.05.

### 3.5 Association Between Network Changes and Behavioral Outcomes

**FIGURE 4:**
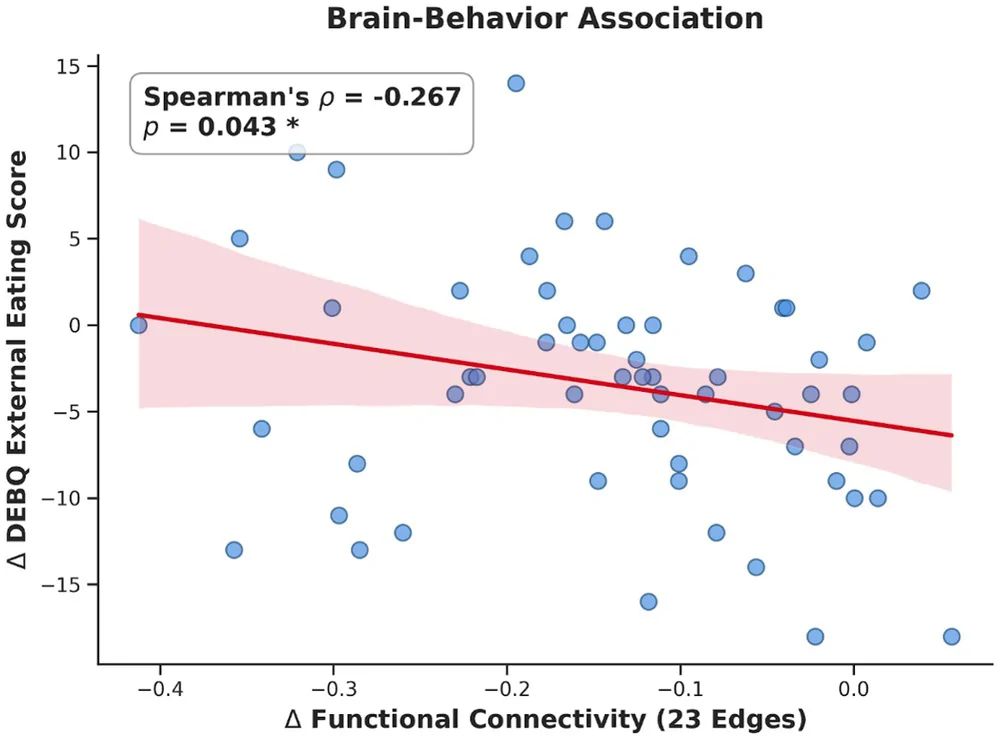
Exploratory association between BN‐derived NBS component connectivity change and external eating change. Scatter plot showing the association between longitudinal change in mean functional connectivity across the 23‐edge BN‐derived NBS component and change in DEBQ‐External score in the BN group. Spearman's rank correlation showed a nominal negative association (*ρ* = −0.267, nominal *p* = 0.043). The displayed *p* value is uncorrected and should be interpreted as exploratory. The red line represents the fitted linear trend, and the shaded area indicates the 95% confidence interval. *Indicates nominal *p* < 0.05. Δ, longitudinal change; BN, bulimia nervosa; DEBQ‐External, Dutch Eating Behavior Questionnaire external eating subscale; FC, functional connectivity; NBS, Network‐Based Statistic.

Spearman's rank correlation analyses showed a modest association between DAN and VAN connectivity change and change in external eating behavior (ΔDEBQ‐External; *ρ* = −0.267, *p* = 0.043; Figure 4). No significant association was observed between DAN and VAN connectivity changes and bulimic symptom frequency (ΔEDI‐BN; *p* = 0.540). In addition, changes in DAN–SMN connectivity were not significantly associated with either behavioral measure (Table 6).

**TABLE 6:** Neurobehavioral specificity of the correlations between functional network decoupling and clinical symptom improvements.

Network‐level FC measure	Clinical symptom change (Δ)	Spearman's *ρ*	*p*
ΔDAN–SMN connectivity	ΔEDI‐BN	−0.081	0.546
ΔDAN–SMN connectivity	ΔDEBQ	−0.053	0.691
ΔDAN–VAN/salience connectivity	ΔEDI‐BN	−0.082	0.540
ΔDAN–VAN/salience connectivity	ΔDEBQ	−0.264	**0.045**

*Note:* FDR correction was performed using the Benjamini–Hochberg procedure across the four exploratory brain–behavior correlations. Nominal *p* values are reported for transparency; associations should be interpreted as exploratory. Bold values indicate nominal *p* < 0.05.

Abbreviations: DAN, dorsal attention network; DEBQ‐external, Dutch Eating Behavior Questionnaire external eating subscale; EDI‐BN, eating disorder inventory bulimia subscale; SMN, somatomotor network; VAN/salience, ventral attention/salience network.

To further evaluate the robustness of the observed association, partial correlation analyses were conducted controlling for potential confounding variables. When controlling for body weight change, the association between DAN–VAN ΔFC and ΔDEBQ‐External remained significant (partial *ρ* = −0.273, *p* = 0.040). However, when controlling for variability in follow‐up duration (ΔTime), the association was attenuated and did not reach conventional statistical significance (partial *ρ* = −0.258, *p* = 0.052). Similarly, when controlling for both ΔBody Weight and ΔTime simultaneously, the association remained at a trend level (partial *ρ* = −0.262, *p* = 0.051; Table 7).

**TABLE 7:** Partial correlation analyses of neurobehavioral associations controlling for clinical covariates.

Variable pair	Covariate controlled	Partial spearman's *ρ*	*p*
Δ FC vs. Δ DEBQ	None	−0.267	**0.043**
Δ FC vs. Δ DEBQ	Δ Body weight	−0.273	**0.040**
Δ FC vs. Δ DEBQ	Δ Time	−0.258	0.052
Δ FC vs. Δ DEBQ	Δ BDI (depression)	−0.218	0.103
Δ FC vs. Δ DEBQ	Δ Body weight and Δ time	−0.262	0.051

*Note:* This table presents Partial Spearman's correlations assessing the robustness of the association between changes in functional connectivity (ΔFC, DAN ↔ VAN) and external eating behavior (ΔDEBQ). The negative association remained significant after controlling for body weight change but was attenuated after controlling for follow‐up interval or change in depressive symptoms (ΔBDI). Bold values indicate nominal *p* < 0.05.

Abbreviations: Δ, longitudinal change; BDI, beck depression inventory; DEBQ, Dutch Eating Behavior Questionnaire; FC, functional connectivity.

## 4 Discussion

In the present longitudinal study, we examined macroscopic functional network changes associated with fluoxetine treatment in patients with BN. Consistent with prior clinical evidence, the intervention was associated with significant improvements in both depressive symptoms and eating‐related behavioral measures [28]. At the neural systems level, within‐BN NBS analysis identified a 23‐edge functional connectivity component showing reduced connectivity after treatment, primarily involving the DAN, VAN/salience, and SMN. Longitudinal healthy‐control reference analyses showed no significant whole‐brain NBS component in either direction. When the BN‐derived component was extracted in both groups, patients with BN showed a marked reduction in component‐level FC, with post‐treatment connectivity shifting toward the healthy‐control reference range. These findings suggest that the observed BN network changes were not simply mirrored by a comparable whole‐brain network‐level change in healthy controls, although the healthy‐control component‐level reduction indicates that nonspecific longitudinal or repeated‐scanning effects should still be considered. Clinically, the modest reduction observed in healthy controls may reflect normal longitudinal variability or reduced novelty and arousal during repeated assessment. In contrast, the greater reduction in BN may indicate attenuation of excessive coupling among attention, salience, and sensorimotor systems involved in externally driven, cue‐responsive eating behavior. Exploratory analyses further revealed a modest, covariate‐sensitive association between DAN and VAN connectivity change and externally driven eating behavior, providing a preliminary and hypothesis‐generating link between attention–salience network dynamics and behavioral improvement in BN.

While internal emotional and homeostatic dysregulation may contribute to the core vulnerability of BN, the disorder is also prominently characterized by heightened sensitivity to external food‐related cues, which may serve as proximal triggers for binge‐eating episodes [29]. From a systems neuroscience perspective, such cue‐driven behavioral tendencies have been linked to altered interactions among large‐scale attentional and salience networks, which support the dynamic balance between goal‐directed cognitive control and stimulus‐driven attentional capture [30]. In the present study, the BN‐derived NBS component included reduced connectivity between the DAN and VAN/salience network during the treatment period. The DAN is commonly implicated in top‐down, goal‐directed attentional allocation, whereas the VAN/salience network is involved in detecting behaviorally relevant or salient stimuli. Altered coordination between these systems has been proposed to contribute to attentional bias toward salient cues, including food‐related stimuli, in eating disorders [31]. Within this framework, the observed reduction in DAN and VAN connectivity may reflect a longitudinal reconfiguration of the interaction between top‐down attentional control and bottom‐up salience processing during treatment. This interpretation is consistent with prior longitudinal neuroimaging evidence suggesting that pharmacological or behavioral interventions in binge‐eating spectrum disorders may be accompanied by changes in large‐scale networks involved in executive control, salience processing, and reward‐related behavior [32].

Post hoc nodal characterization indicated prominent involvement of the right temporo‐occipito‐parietal parcel (Node 148) and the right somatomotor parcel (Node 118) within the BN‐derived NBS component. These findings help localize the significant network to attention‐, salience‐, and sensorimotor‐related systems. This interpretation is consistent with recent network accounts suggesting that the salience network supports the allocation of neural resources according to salience and cognitive demand [33], as well as eating‐disorder neuroimaging evidence implicating altered responses to food‐related stimuli and disrupted large‐scale functional connectivity in eating‐disorder psychopathology [34, 35].

In this context, the concurrent involvement of VAN/salience and SMN parcels suggests that the observed treatment‐period‐associated connectivity reductions were organized around systems relevant to cue detection, salience processing, and sensorimotor integration.

Exploratory brain‐behavior analysis showed a nominal association between DAN and VAN connectivity change and change in DEBQ‐External scores, a dimension reflecting eating in response to external food‐related cues [36], whereas no comparable association was observed with ΔEDI‐BN. This pattern is broadly consistent with recent evidence that individuals with binge‐eating behaviors show heightened attentional bias toward high‐calorie food cues, suggesting that cue‐related attentional processing may be particularly relevant to externally driven eating tendencies [37]. However, this association was modest in magnitude and was attenuated after controlling for follow‐up interval or depressive symptom change, indicating that it should not be interpreted as evidence for an independent or causal pathway. Given that fluoxetine has established clinical efficacy in BN and may also affect mood‐related symptoms [38], improvement in depressive symptoms may partly contribute to behavioral recovery during the treatment period. Therefore, the present findings provide preliminary, hypothesis‐generating evidence that treatment‐period‐associated attention‐salience network changes may be related to externally driven eating behavior, but this relationship requires confirmation in larger controlled longitudinal studies.

Several limitations should be acknowledged. First, although the longitudinal healthy‐control cohort helped account for nonspecific repeated‐scanning and temporal effects, the absence of a placebo‐treated BN group precludes definitive attribution of the observed network changes to fluoxetine‐specific effects. Second, the sample size remains limited for detecting modest brain–behavior associations, particularly after covariate adjustment; therefore, the DAN–VAN–DEBQ–External association should be interpreted as exploratory. Third, the nodal analyses were post hoc characterizations of the BN‐derived NBS component and were intended to localize the significant network rather than to identify independent pathological hubs. Finally, resting‐state fMRI cannot directly capture food‐cue reactivity, craving states, or real‐time binge‐eating dynamics. Future randomized controlled studies combining longitudinal neuroimaging with task‐based food‐cue paradigms and ecological momentary assessment are needed to clarify the clinical relevance and mechanistic specificity of these network changes.

## 5 Conclusion

In conclusion, this longitudinal rs‐fMRI study showed that the fluoxetine treatment period in patients with BN was accompanied by clinical and behavioral improvement, together with reduced functional connectivity within a BN‐derived network involving the DAN, VAN/salience, and SMN. Whole‐brain longitudinal NBS analysis in healthy controls did not identify a significant connected component. When the BN‐derived 23‐edge component was examined relative to the healthy‐control reference cohort, follow‐up connectivity in BN shifted toward the healthy‐control reference range. Exploratory brain‐behavior analyses further suggested a modest association between DAN–VAN connectivity change and externally driven eating behavior. These findings provide preliminary systems‐level evidence that large‐scale functional network changes accompany clinical improvement during fluoxetine treatment in BN, and warrant confirmation in randomized controlled longitudinal studies.

## Author Contributions

Y.J. and W.L. are co‐first authors who contributed equally to the study design, execution, data analysis, and manuscript drafting. Z.W., L.T., and P.Z. are co‐corresponding authors who jointly supervised the project, conceived the original idea, and critically revised the manuscript. Specifically, Y.J. and W.L. led the neuroimaging processing. F.Y., Y.W., and J.W. managed patient recruitment, clinical assessments, and sample collection. G.W. provided technical assistance with MRI acquisition and quality control. P.Z. is the guarantor of this work and takes responsibility for the integrity of the data and the accuracy of the data analysis. All individuals listed meet the criteria for authorship, and all those who qualify for authorship are included. All authors read and approved the final manuscript.

## Funding

This research was funded by Grants 82572162, 82001790, and 82502308 from the National Natural Science Foundation of China, the Cultivation Fund of Capital Medical University (PYZ24061), Beijing Key Clinical Discipline Funding (No. 2021135), and the Beijing Key Laboratory of Mental Disorders (2021JSJB03).

## Ethics Statement

The Institutional Review Board of Beijing Friendship Hospital, Capital Medical University approved this single‐center prospective cohort study (approval number: 2021‐P2‐052‐01).

## Conflicts of Interest

The authors declare no conflicts of interest.

## Data Availability

The data that support the findings of this study are available from the corresponding author upon reasonable request.
